# Discovering Hidden Archaeal and Bacterial Lipid Producers in a Euxinic Marine System

**DOI:** 10.1111/1462-2920.70054

**Published:** 2025-02-27

**Authors:** Dina Castillo Boukhchtaber, F. A. Bastiaan von Meijenfeldt, Diana X. Sahonero Canavesi, Denise Dorhout, Nicole J. Bale, Ellen C. Hopmans, Laura Villanueva

**Affiliations:** ^1^ Department of Marine Microbiology and Biogeochemistry Royal Netherlands Institute for Sea Research t Horntje the Netherlands; ^2^ Faculty of Sciences. Department of Biology Utrecht University Utrecht the Netherlands

**Keywords:** (genome‐resolved) metagenomics, Black Sea, branched GDGTs (brGDGTs), glycerol dialkyl glycerol tetraethers (GDGT), high‐resolution accurate mass spectrometry, isoprenoid GDGT (isoGDGTs), membrane lipids, membrane‐spanning lipid synthase, membrane‐spanning lipids, tetraether synthase

## Abstract

Bacterial membrane lipids are typically characterised by fatty acid bilayers linked through ester bonds, whereas those of Archaea are characterised by ether‐linked isoprenoids forming bilayers or monolayers of membrane‐spanning lipids known as isoprenoidal glycerol dialkyl glycerol tetraethers (isoGDGTs). However, this understanding has been reconsidered with the identification of branched GDGTs (brGDGTs), which are membrane‐spanning ether‐bound branched alkyl fatty acids of bacterial origin, though their producers are often unidentified. The limited availability of microbial cultures constrains the understanding of the biological sources of these membrane lipids, thus limiting their use as biomarkers. To address this issue, we identified membrane lipids in the Black Sea using high‐resolution accurate mass/mass spectrometry and inferred their potential producers by targeting lipid biosynthetic pathways encoded on the metagenome, in metagenome‐assembled genomes and unbinned scaffolds. We also identified brGDGTs and highly branched GDGTs in the suboxic and euxinic waters, potentially attributed to Planctomycetota, Cloacimonadota, Desulfobacterota, Chloroflexota, Actinobacteria and Myxococcota—based on their lipid biosynthetic genomic potential. These findings introduce new possibilities for using specific brGDGTs as biomarkers of anoxic conditions in marine environments and highlight the role of these membrane lipids in microbial adaptation.

## Graphical abstract

We investigated the bacterial and archaeal membrane lipids in the Black Sea, focusing on isoprenoidal and branched GDGTs, combining high‐resolution accurate mass/mass spectrometry with metagenomics. Our objective was to identify the potential biological producers of these lipids and to begin understanding their role in microbial adaptation.

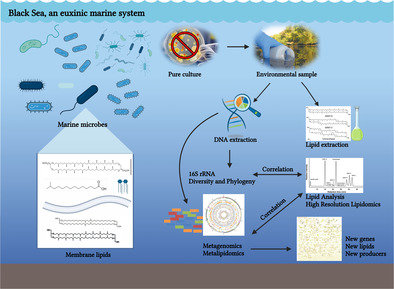

## 1 Introduction

Microbial membrane lipids are the main components of the cell membrane, which are either composed of fatty acids linked through ester bonds to glycerol‐3‐phosphate (G3P) in the case of bacteria, or of isoprenoids linked through ether bonds to glycerol‐1‐phosphate (G1P) in archaea. Archaeal membranes can be organised as bilayers of diether lipids or as monolayers of tetraethers or so‐called isoprenoid glycerol dialkyl glycerol tetraethers (isoGDGTs) (Koga and Morii 2007; Villanueva, Damsté, and Schouten 2014). Conversely, bacterial membranes are generally composed of bilayers. Exceptions are bacterial membrane‐spanning lipids (MSLs) composed of branched alkyl chains derived from fatty acids (FAs) and ether bonds, or so‐called branched GDGTs (brGDGTs), which display characteristics typically found in archaeal membrane lipids, that is, ether bonds and a membrane‐spanning nature (Schouten, Hopmans, and Sinninghe Damsté 2013). Microbial membrane lipids can be used as taxonomic markers for specific microbial groups when found in the environment (Damsté et al. 2000; Hamersley et al. 2007; Rush and Sinninghe Damsté 2017). Furthermore, microbial membrane lipids may change as a response to environmental changes to maintain the homeostasis of the membrane, thus also providing information on the physiological status of the cell (Sohlenkamp and Geiger 2016). Lipids can be preserved in the sedimentary record longer than most other biomolecules like DNA or proteins, which makes them potential biomarkers for the presence of specific microbial groups or environmental conditions in the past (Brocks and Pearson 2005; Rush and Sinninghe Damsté 2017). A known example of well‐preserved lipids in the sedimentary record widely used as proxies to reconstruct past environmental conditions are microbial (iso and br) GDGTs (Pearson and Ingalls 2013; Schouten, Hopmans, and Sinninghe Damsté 2013; Zhao et al. 2023; Otiniano et al. 2024). Isoprenoid GDGTs are known to be synthesised by several archaeal phyla in soils, freshwater, and in the marine environment (Schouten, Hopmans, and Sinninghe Damsté 2013; Figure S1). Branched GDGTs are found in soils, peats and in freshwater systems (Pancost and Sinninghe Damsté 2003; Weijers et al. 2006, 2007) and have also been detected in marine oxygen minimum zones (Liu et al. 2014) suggesting multiple bacterial source types and physiologies producing these lipids in the environment. Members of the bacterial phylum Acidobacteriota have been seen to synthesise the building blocks of brGDGTs (i.e., iso‐diabolic acid; (Sinninghe Damsté et al. 2011; Chen et al. 2022; Halamka et al. 2023)) but in general the bacterial sources of brGDGTs are unknown. Recent genomic analyses based on the presence of biosynthetic genes suggest that multiple bacterial, and potentially even archaeal groups, might be able to synthesise brGDGTs (Sahonero‐Canavesi et al. 2022).

To constrain the biological source(s) of GDGTs or any other lipid, the ultimate proof is the isolation of the producer and confirmation of the lipid production in the laboratory. However, only a small percentage of microorganisms have been cultured in the lab (Tyson and Banfield 2005; Overmann, Abt, and Sikorski 2017; Lewis et al. 2021) thus preventing this approach in most cases. Alternatively, it is possible to investigate the genetic capacity of a microorganism to synthesise a lipid compound of interest in the environment without lab culture by detecting the genes coding for enzymes involved in the production of these lipids (Pearson et al. 2007; Welander et al. 2010). Most of the enzymes involved in the isoGDGT biosynthetic pathway are known (Koga and Morii 2007; Jain et al. 2014); recently, several key steps in the pathway have been elucidated, such as the formation of the monolayer by coupling of the terminal‐ends of the isoprenoids by the radical S‐adenosyl‐L‐methionine (SAM) enzyme Tes (tetraether synthase; Lloyd et al. 2022; Zeng et al. 2022) as well as the formation of cyclopentane rings mediated by radical SAM GDGT ring synthases (gyrases GrsAB; Zeng et al. 2019). A recent study has also suggested the involvement of a radical SAM protein, a glycerol monoalkyl glycerol tetraether (GMGT) synthase (Gms), mediating the formation of a covalent bond between the two isoprenoid chains in isoGDGTs to form GMGT (previously known as H‐GDGTs; Garcia et al. 2024; Li et al. 2024). On the other hand, the lipid biosynthetic pathway of brGDGTs seems to be more diverse and is not yet fully constrained. Recently, we identified the MSL synthase (Mss) and glycerol ester reductase (Ger), involved, respectively, in the coupling of two FAs and the conversion of ester to alkyl ether bonds in brGDGTs mostly under anaerobic conditions (Sahonero‐Canavesi et al. 2022). Homologues of Tes that are found in bacterial genomes have also been proposed to be involved in the coupling of fatty acids in the formation of brGDGTs, however, the proposed enzymatic activity for these enzymes has not been confirmed (Zeng et al. 2022). Moreover, the enzymes encoded by the ether lipid biosynthesis gene cluster *elb* (i.e., elbB, D and E) in myxobacteria have been seen to be involved in the formation of alkyl ethers (Lorenzen et al. 2014), thus suggesting multiple potential ways to synthesise ether bonds in membrane lipids. Additionally, an alternative pathway involving an alkylglycerone phosphate synthase encoded by the *agps* gene is involved in the formation of alkenyl ethers (plasmalogens) in *Myxococcus xanthus* (Lorenzen et al. 2014) while alkenyl ethers can also be synthesised by the products of the *pls* operon under anaerobic conditions (Jackson et al. 2021). Even though specific microbial groups have been predicted to make iso and brGDGTs based on the presence of the above‐mentioned genes in their genomes, constraining the producers in environmental samples is essential for making better interpretations of their use as biomarkers of paleoenvironmental proxies, their adaptive value to the microorganism, as well as to elucidate the timing of the evolutionary acquisition of these membrane lipids.

Here, we investigated the microbial sources of membrane‐spanning lipids (MSL, iso and brGDGTs) by using genome‐resolved metagenomics targeting their lipid biosynthetic pathways in the Black Sea, the biggest euxinic (sulfidic and anoxic) basin in the world, where both iso and brGDGTs have been previously detected (Schouten et al. 2000; Liu et al. 2014). In addition, we report the brGDGTs composition and distribution in the Black Sea water column and compare them with microbial MSL potential producers to better constrain the sources of these lipids. Data derived from this study will also further aid in the cultivation and validation of the production of iso and brGDGTs of microorganisms present in the marine environment.

## 2 Results and Discussion

In this study, we investigate the isoGDGT lipid data by Sollai et al. (2019) and we also report brGDGT lipid data obtained from the same samples of the Black Sea water column from 50 to 2000 m depth—which were extracted and analysed with a different approach (see Section 4 for details). In addition, DNA was extracted from the same samples and 16S rRNA gene amplicon sequencing, 16S rRNA gene quantification using quantitative PCR, and genome‐resolved metagenomics (previously reported in Ding and von Meijenfeldt et al. 2004) were performed to determine the taxonomy, abundance and lipid biosynthetic pathways of potential iso‐ and brGDGTs producers in this system.

### 2.1 Physicochemical Conditions, General Microbial Diversity and Abundance in the Black Sea Water Column

Suspended particulate matter (SPM) samples had been collected from the Black Sea water column in high resolution from 50 to 2000 m depth. Oxygen and sulfide concentrations were previously reported in Sollai et al. (2019) and summarised in Figure S2. In brief, oxygen concentration decreased from 50 to 70 m depth, with suboxic waters from 70 to 110 m depth, and euxinic waters spanning down to 2000 m depth where oxygen was below the detection limit and the sulfide concentration was approximately 400 μM (Figure S2).

Microbial diversity was evaluated by 16S rRNA gene amplicon sequencing using universal primers to capture a broad phylogenetic diversity. The same 16S rRNA gene primers were used for estimating 16S rRNA gene absolute abundances attributed to different archaeal and bacterial groups based on quantitative PCR (see Section 4 for details).

Bacterial 16S rRNA gene reads were predominant throughout the water column, with values higher than 80% of the total (bacteria + archaea + unassigned at the domain rank) except for the depths between 250 and 1000 m where bacterial relative abundance was lower than 70% (Table S1). Archaeal 16S rRNA gene reads ranged from 1% to 11% of the total throughout the depth profile, with maximum relative abundance in the suboxic zone (70–100 m), which then decreased to values around 2% in the euxinic zone (Table S1).

**FIGURE 1:**
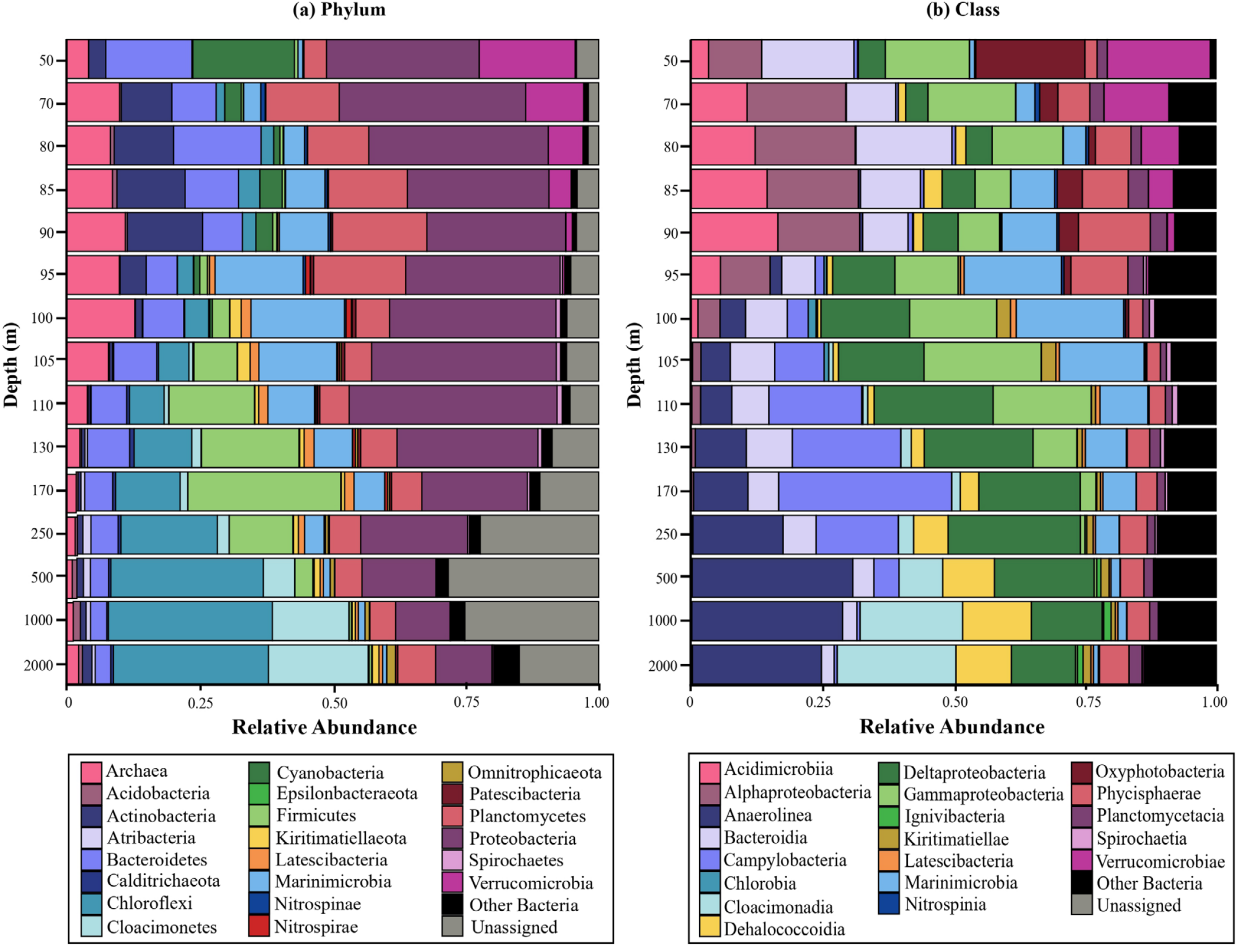
General bacterial diversity at the (a) phylum and (b) class rank based on 16S rRNA gene amplicon sequencing data in the Black Sea water column.

Bacterial diversity profiles based on 16S rRNA gene amplicon sequencing revealed a predominance of Bacteroidetes, Cyanobacteria, Proteobacteria and Verrucomicrobia in the oxic waters (i.e., 50 m depth) (Figure 1, Table S2A). In suboxic waters (i.e., 70–110 m depth), the 16S rRNA gene amplicon sequencing profiles indicated the major presence of the phyla Actinobacteria (mainly class Acidimicrobiia, 12%), Bacteroidetes (mainly class Bacteroidia, up to 16% at 80 m), Epsilonbacteraeota (mainly class Campylobacteria, up to 30% at 170 m), Planctomycetota (up to 15%) and Marinomicrobia (up to 16%). Sequences attributed to the phylum Proteobacteria were also present in the suboxic waters, with the classes Alphaproteobacteria predominant in the upper suboxic waters (up to 15%), Gammaproteobacteria in the lower suboxic waters (up to 20%), and Deltaproteobacteria in the lower suboxic waters (up to 18%), also extending their presence in the euxinic waters (Figure 1, Table S2A). Some of the Deltaproteobacteria 16S rRNA gene sequences at these depths can be attributed to sulphate‐reducing bacteria (Table S2A), which fits with a relevant role and presence of anaerobic organic matter processing sulphate reducers in the lower suboxic and euxinic waters of the Black Sea (Vliet et al. 2021). Finally, bacterial 16S rRNA gene reads in the euxinic waters were dominated by members of the phylum Chloroflexi, (specifically classes Anaerolineae and Dehalococcoidia) increasing in abundance, from 130 m downwards, −up to 30% of all reads; members of the phylum Cloacimonadota (prev. ‘*Candidatus* Cloacimonetes’) only present from 500 m depth downwards and to a maximum of 14% between 1000 and 2000 m (Figure 1, Table S2A). The here‐reported bacterial diversity coincides with previously reported studies in the Black Sea water column in different geographical stations, years and seasons (Cabello‐Yeves et al. 2021; Pavlovska et al. 2021; Suominen et al. 2021), which suggests that the microbial population is highly stable and that this system allows a comprehensive study with conclusions applicable to larger spatial and temporal scales.

**FIGURE 2:**
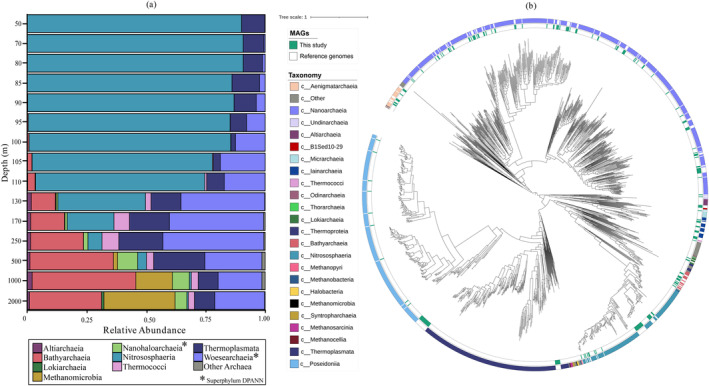
General archaeal diversity based on (a) 16S rRNA gene amplicon data (at the class rank), and (b) phylogenetic tree rooted at mid‐point, with the metagenome‐assembled genomes (MAGs) from the Black Sea (this study) in green and reference genomes in white, taxonomically classified according to the GTDB‐Tk).

Archaeal diversity profiles based on 16S rRNA gene amplicon sequencing indicated that the phylum Thaumarchaeota (Nitrososphaerota), specifically the class Nitrososphaeria, constituted 85%–90% of total archaeal 16S rRNA gene reads in suboxic waters and then the percentage declined downward (Figure 2, Table S1). Members of the class Bathyarchaeia (phylum Crenarchaeota) contributed on average 40% of total archaeal 16S rRNA gene reads from 250 to 2000 m, while class Thermoplasmata (phylum Euryarchaeota) were present in the entire water column, with those Thermoplasmata affiliated to Marine Euryarchaeota group II abundant in the oxic and suboxic waters, whereas those attributed to uncultured Thermoplasmatales mostly present in euxinic waters (Figure 2A, Table S2A). Sequences attributed to the class Woesearchaeia (phylum Nanoarchaeota, DPANN superphylum) contributed up to 40% of the total archaeal 16S rRNA gene reads in the upper euxinic waters but were also present in suboxic and euxinic waters (Figure 2A, Table S2A). The DPANN superphylum is an acronym formed by the initials of the first five groups discovered, Diapherotrites, Parvarchaeota, Aenigmarchaeota, Nanoarchaeota and Nanohaloarchaeota, Because of their genome size and metabolic capacities, there are several DPANN phyla described to have symbiotic lifestyles and depending on either an archaeal or bacterial host (Castelle et al. 2018; Dombrowski et al. 2019). In general, the archaeal diversity reported here by using 16S rRNA gene amplicon sequencing is similar to that previously reported in the same samples with different sequencing primers and a lower amount of sequenced reads by using another sequencing platform (Sollai et al. 2019).

Nevertheless, due to deeper sequencing efforts, new archaeal groups were detected in the current study, including members of the class Altiarchaeia (phylum Altiarchaeota) and of the order Aenigmarchaeales (class Nanohaloarchaei, part of the phylum Nanoarchaeaeota, DPANN superphylum), which accounted for approximately 2% and 7% of the total archaeal 16S rRNA reads in the euxinic waters, respectively (Table S2A).

Total estimated archaeal abundance based on quantitative PCR showed a different distribution to that observed by Sollai et al. (2019) in the same samples, showing the highest abundance of archaeal 16S rRNA gene reads at 85 m depth at the upper interface of the suboxic zone (Figure S3, Table S3), while in the previous study, the maximum abundance was detected well within the suboxic zone (70–100 m), showing the discrepancies that can be induced by using different 16S rRNA gene amplification primers on the same samples (e.g., Tremblay et al. 2015). Bacterial abundance estimated as total bacterial 16S rRNA gene copies per litre of SPM indicated maximum values at 85 m depth (Figure S3, Table S3). Maximum bacterial abundance in the euxinic waters was detected at 1000 m coinciding with the maximum of archaeal 16S rRNA gene copies L^−1^ but one order of magnitude higher than those attributed to archaea (Figure S3, Table S3).

Archaeal diversity was further investigated by determining the diversity of metagenome‐assembled genomes (MAGs) classified as Archaea that were obtained by sequencing the metagenome followed by de novo assembly and binning of the scaffolds of the same extracted DNA used for the amplicon sequencing (Ding and von Meijenfeldt et al. 2004; see Section 4 for details). MAGs affiliated with the phyla Aenigmatarchaeota, Altiarchaeota, Asgardarchaeota, Hadarchaeota and Ianarchaeota. Micrarchaeota, Nanoarchaeota (e.g., of orders Woesearchaeales and Pacearchaeales), Thermoplasmatota (of classes Poseidoniia and Thermoplasmata), Thermoproteota (of class Bathyarchaeia and order Nitrososphaerales) and Undinarchaeota were detected (classified based on GTDB‐Tk, see Table S4). To further visualise this wide taxonomic diversity detected in the Black Sea, we generated a phylogenetic tree of the archaeal MAGs. MAGs were dereplicated and further selected based on their completeness (≥ 50%) and used to build a phylogenetic tree with a selection of known archaeal genomes based on a concatenated alignment of 24 core vertically‐transferred genes (Figure 2B; see Section 4 for details). The archaeal taxonomic groups detected by metagenomic sequencing were largely similar to the groups detected in this study by 16S rRNA gene amplicon sequencing (Figure 2A), indicating that the 16S rRNA gene primers used in this study are not considerably taxonomically biased. Some minor inconsistencies exist between the taxonomic profiles generated via amplicon sequencing and the taxonomic groups identified with metagenomic sequencing, which arise because of the use of different taxonomic analysis pipelines. For example, Bathyarchaeia are considered part of the phylum Crenarchaeota in our 16S rRNA gene amplicon sequencing analysis, which is considered the phylum Thermoproteota in our metagenomes. For Bacteria, reads from the phylum Desulfobacterota, relevant for most of the metagenomic analysis of this article, correspond to reads of what was previously phylum Proteobacteriota (class Deltaproteobacteria, order Desulfobacterales) in the 16S rRNA analysis. The complete correspondence between the two analyses and the disparities in classification can be consulted in Table S2B. For the remaining text, figures and tables, we will be using the CAT (Contig Annotation Tool) and BAT (Bin Annotation Tool) taxonomic classifications of the scaffolds and MAGs.

### 2.2 Membrane Lipid Diversity in the Black Sea Water Column

#### 2.2.1 Isoprenoid GDGTs Diversity and Distribution, and Potential Producers

**FIGURE 3:**
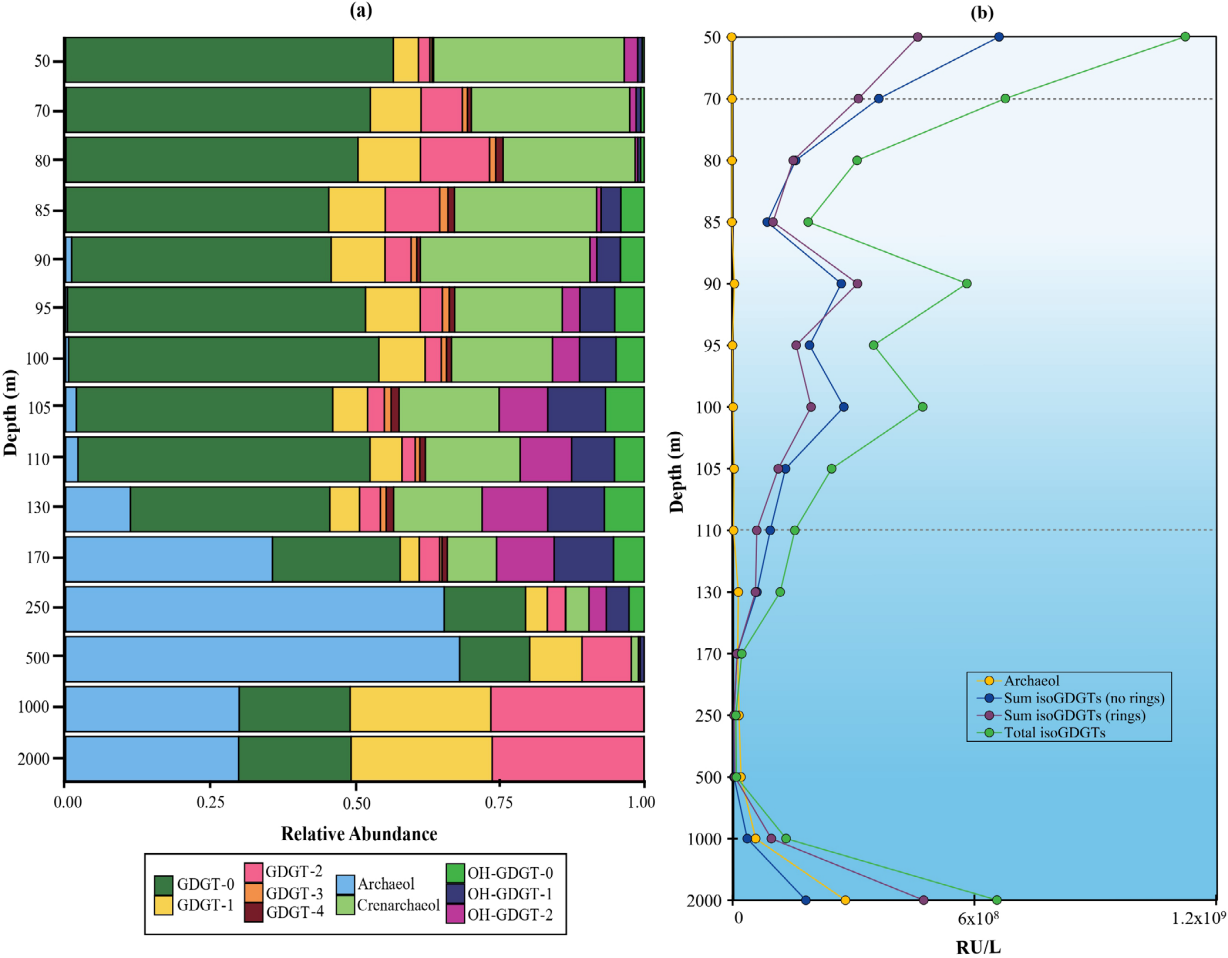
Distribution of archaeal isoGDGTs and archaeol (sum of intact polar lipid‐derived core lipids reported by Sollai et al. 2019) in the Black Sea water column, (a) relative abundance, (b) response units (RU) per litre of isoGDGTs with no rings (i.e., GDGT‐0), with rings and total isoGDGTs. Dotted lines indicate the beginning of suboxic (70 m) and euxinic (110 m) zones.

Here, we used the isoGDGT data previously reported by Sollai et al. (2019) represented as the sum of all the intact polar lipid (IPL)‐derived core lipids present in the water column at a determined depth. The distribution of archaeal lipids, that is, archaeol and isoGDGTs, in the water column showed that archaeol was mostly present in deep euxinic waters, while isoGDGTs were abundant in the surface (50 m), suboxic, and in the deep euxinic waters (Figure 3A, Table S5). Few archaeal lipids were present at 170–500 m depth (Figure 3B). Although both isoGDGTs with no rings (i.e., GDGT‐0) and with rings (GDGT‐1‐4 and crenarchaeol) had similar vertical distribution, the presence of isoGDGTs with rings increased with depth in comparison with the surface water where GDGT‐0 was predominant (Figure 3B).

Sollai et al. (2019) suggested that the lipid composition in the oxic and suboxic waters was likely mainly attributed to members of the Thaumarchaeota and the Marine Euryarchaeota group II, whereas the lipids detected in the deep euxinic waters could be synthesised by archaea belonging to the DPANN superphylum, as well as classes Thermoplasmata, Bathyarchaeia and members of ANME‐1b, which were suggested to be responsible for the synthesis of archaeol, GDGT‐0, GDGT‐1 and GDGT‐2. The observed GDGT‐1 and 2 in the euxinic waters of the Black Sea have been traditionally thought to be produced in situ by members of the methanotrophic ANME group (e.g., Wakeham et al. 2003; Blumenberg et al. 2004). Although molecular carbon isotope evidence has supported the transfer of isotopically depleted methane‐derived carbon into lipids of ANME archaea in the deep euxinic waters of the Black Sea, the ^13^C values measured in the bulk GDGT building blocks biphytanes have been previously reported to be substantially higher than observed in most other environments containing anaerobic methanotrophic archaea (Wakeham et al. 2003). An explanation for this offset might be additional or alternative sources to ANME‐1 for isoGDGTs with rings in the deep euxinic waters of the Black Sea. The study by Sollai et al. (2019) showed that other archaeal groups like members of the order Thermoplasmatales (phylum Thermoplasmatota) and of the class Bathyarchaeia (phylum Thermoproteota) outnumbered ANME‐1 at these depths, so potentially these groups could be also synthesising isoGDGTs with rings and therefore invalidate the use of GDGT‐1 and 2 as exclusive biomarkers of anaerobic methane oxidizers in similar marine systems. Amongst these archaeal groups, the presence of Bathyarchaeia and Thermoplasmatales in the deep euxinic waters is surprising, as these are archaeal groups normally found in sediments rather than in the water column. Their membrane lipid composition has never been assessed but previous studies have inferred the synthesis of GDGT‐0 (Buckles et al. 2013) or butanetriol dialkyl glycerol tetraethers (BDGTs) (Meador et al. 2014) in members of the Bathyarchaeia based on the co‐occurrence of lipids and these archaeal groups. However, BDGTs have not been detected in these samples (Sollai et al. 2019; this study), suggesting these are not produced by the Bathyarchaeia subgroups present in the Black Sea water column at the time of sampling. The membrane lipid composition of yet‐uncultured sedimentary Thermoplasmatales has never been assessed; therefore, their potential of synthesising GDGTs, with or without ring moieties, is uncertain. This is also applicable to the abundant archaeal DPANN superphylum (classes Nanohaloarchaeia and Woesearchaeia, Figure 2a) present in the euxinic waters (Sollai et al. 2019; this study). Nevertheless, some of the main DPANN groups present in the deep Black Sea are known to have very streamlined genomes lacking lipid biosynthetic capabilities (Castelle et al. 2018; Dombrowski et al. 2019, 2020), so it is likely they do not directly contribute to the GDGT pool in the Black Sea water column.

#### 2.2.2 Branched GDGTs Diversity, Distribution and Potential Producers

**FIGURE 4:**
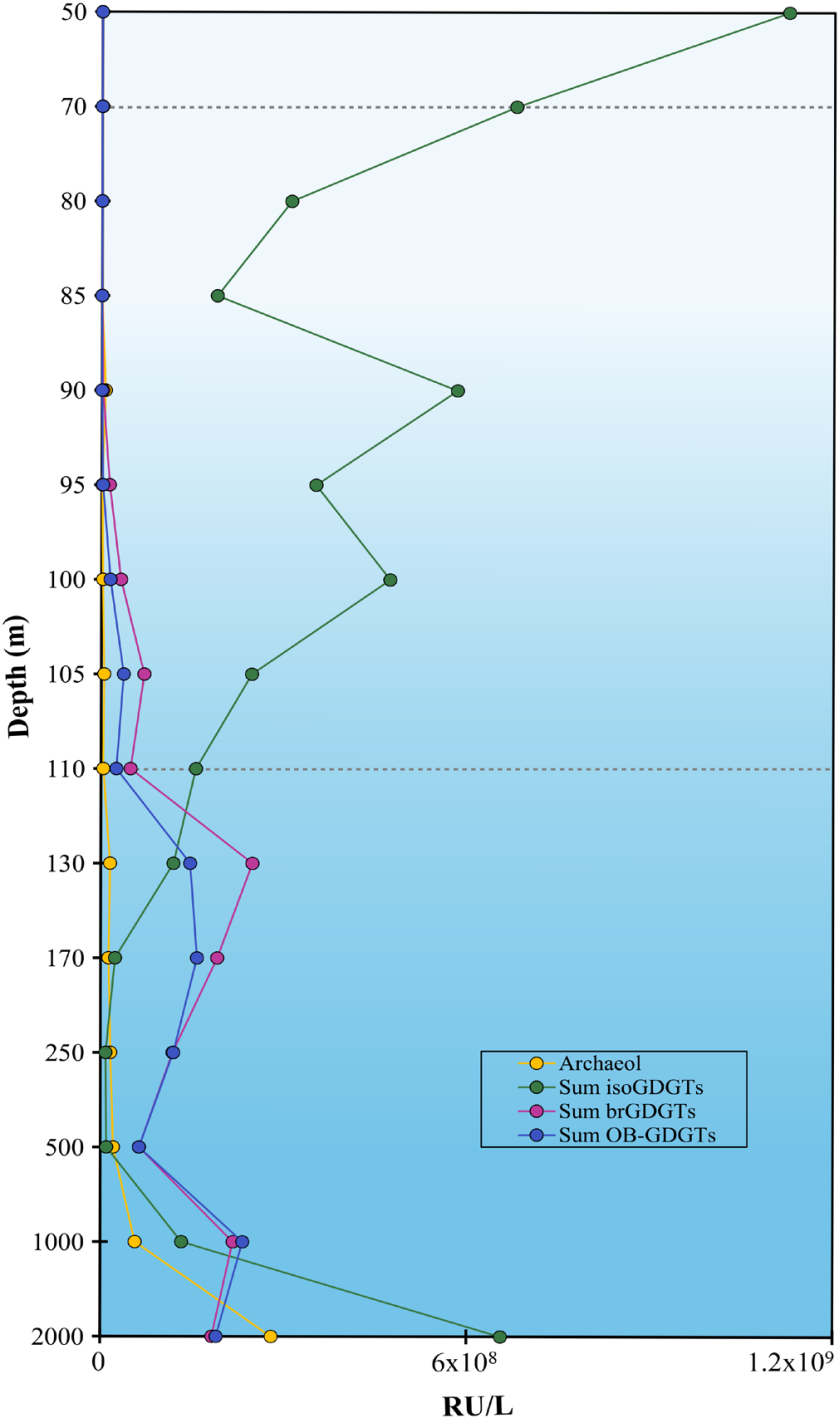
Distribution of the sum of isoGDGTs (intact polar lipid‐derived core lipids), branched GDGTs (brGDGTs, core lipids) and overly branched GDGTs (OB‐GDGTs, core lipids), as response units (RU) per litre in the Black Sea water column. Dotted lines indicate the beginning of suboxic (70 m) and euxinic (110 m) zones.

Previous studies have reported the presence of brGDGTs and overly‐branched GDGTs with a higher degree of methylation (Weijers et al. 2006, 2007; De Jonge et al. 2015; Zeng et al. 2023) in oxygen minimum zones (Xie et al. 2014), in surface sediments (e.g., Liu, Summons, and Hinrichs 2012b; Xie et al. 2014; Becker et al. 2015) and in lake sediments (e.g., Tyler et al. 2010; Niemann et al. 2012; Günther et al. 2014), as well as in the Black Sea water column (Schouten et al. 2000; Liu et al. 2014), which have been attributed to anaerobic planktonic microorganisms (Liu et al. 2014; Xie et al. 2014). An increase in the relative abundance of OB‐GDGTs has been observed in sediments representing the Oceanic Anoxic Event 2, suggesting OB‐GDGTs could be used to infer past marine anoxic conditions (Connock, Owens, and Liu 2022). To better assess the (paleo) proxy potential of these lipids, in‐depth studies of their biological sources and their functional role are needed. In our study, both regular brGDGTs and OB‐GDGTs were detected in the Black Sea water column as previously described (Liu et al. 2014), although they were outnumbered by the isoGDGTs from 130 to 1000 m depth (Figure 4). Distribution of isoGDGTs, brGDGTs and OB‐GDGTs was similar to that previously detected by Liu et al. (2014) in the Black Sea, with isoGDGTs being more abundant in the upper redoxcline, and brGDGTs and OB‐GDGTs increasing in concentration with depth (Figure 4). Nevertheless, the brGDGT distribution by Liu et al. (2014) reported a consistent increase of these lipids with depth up to 2000 m, while in our samples, the distribution follows a less consistent pattern, with an increase observed until 130 m and then again at 1000 m and decrease both at 110 and 500 m and subsequently at 2000 m (Figure 4).

In general, previous studies and ours report a similar distribution of iso‐, br‐ and OB‐GDGTs in the water column of the Black Sea, reinforcing the idea that this euxinic system is very stable in terms of microbial diversity and producers of membrane lipids, over different years and seasons.

**FIGURE 5:**
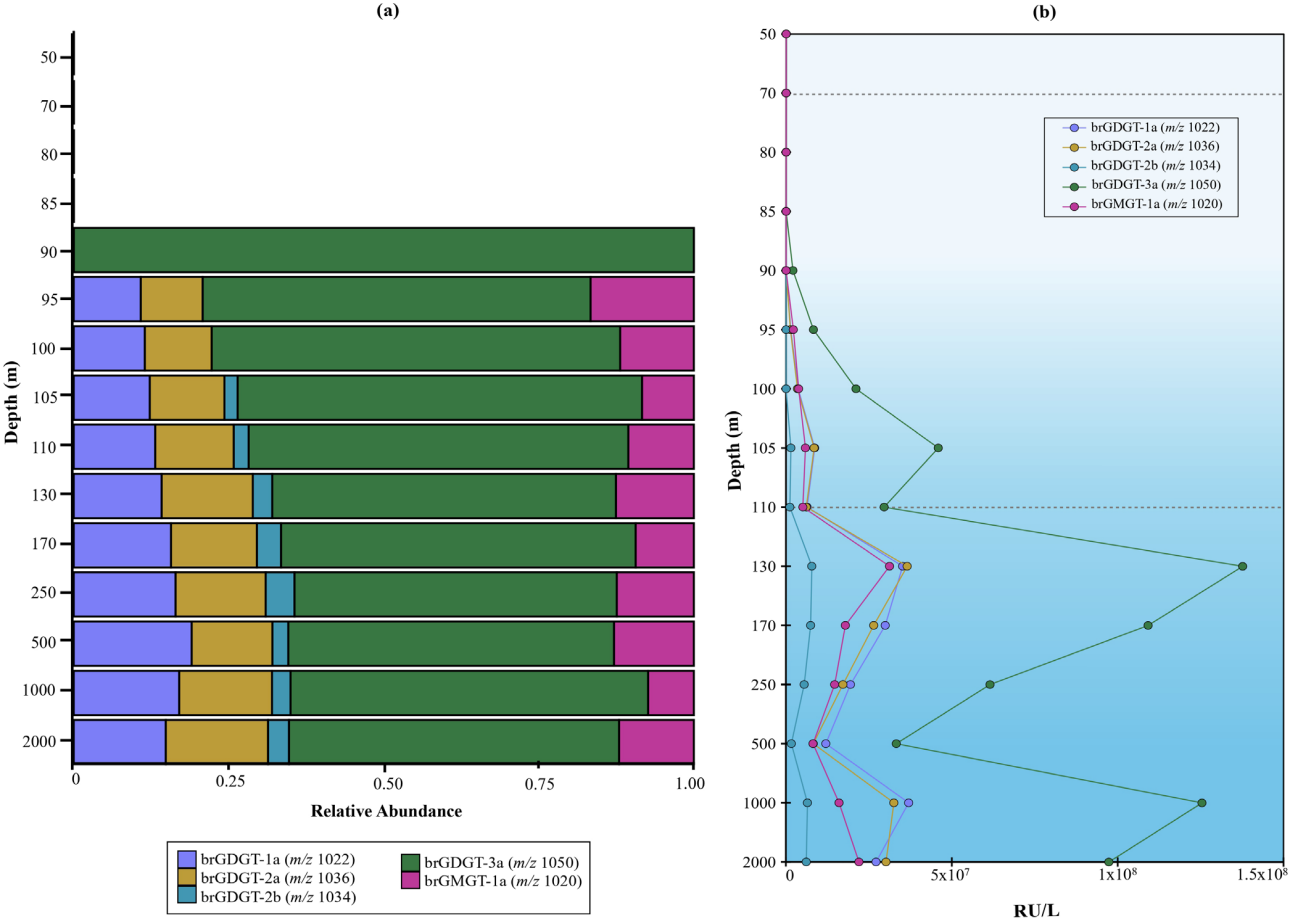
Distribution of branched GDGTs in the Black Sea water column, (a) relative abundance, (b) response units per litre. Dotted lines indicate the beginning of suboxic (70 m) and euxinic (110 m) zones.

In our study, we identified the brGDGTs and OB‐GDGTs based on their accurate mass and elution patterns, however, for an easier visualisation, we will refer to the identified compounds by their nominal mass, both in the text and figures (for more details, including the characteristic fragmentation (CF) observed, see Table S7). We detected brGDGTs ‐1a (mass‐to‐charge, *m/z* 1022; Figure S4A), ‐2a (*m/z* 1036, Figure S4B), ‐2b (*m/z* 1034, Figure S4D) and ‐3a (*m/z* 1050, Figure S4C). Only brGDGTs ‐1a (*m/z* 1022), ‐2a (*m/z* 1036), and ‐3a (*m/z* 1050) were reported previously by Liu et al. (2014) in the Black Sea water column, with brGDGT‐3a (*m/z* 1050) the most abundant from the suboxic waters downwards. The same distribution was observed in our study as brGDGT‐3a (*m/z* 1050) was dominant in the euxinic waters followed by brGDGT‐1a (*m/z* 1022) and ‐2a (*m/z* 1036) (Figure 5A).

The results indicated that all the detected brGDGTs had a similar distribution with local abundance maxima at 105, 130 and 1000 m depth (Figure 5B). Nevertheless, these detected abundance peaks were higher for brGDGT‐3a (*m/z* 1050) than for the other brGDGTs, suggesting the potential contribution of additional producers of this brGDGT at that specific depth or specific environmental conditions that favor the production of brGDGT‐3a.

We also detected the glycerol monoalkyl glycerol tetraether lipid (GMGT; also called ‘H‐GDGTs’) brGMGT‐1a (*m/z* 1020) (Figure S4E) with a maximum abundance at 130 m depth, followed by a decrease with depth, and then an increase from 1000 to 2000 m depth This was the only brGDGT that was higher in abundance at 2000 m than 1000 m depth (Figure 5B).

Oxygen limitation is likely to trigger brGDGT production (Halamka et al. 2021), in terrestrial environments branched GMGTs are generally present at sites characterised by high nutrient levels and/or oxygen‐limited conditions, suggesting anaerobic microbes as likely sources (Naafs et al. 2018; Elling et al. 2023). In the marine realm, brGMGT‐1a (*m/z* 1020) has been detected in marine surface sediments (Liu et al. 2012a), in suspended particulate matter from the oxygen minimum zone of the eastern Pacific (Xie et al. 2014), and in the Bay of Bengal (Kirkels, Usman, and Peterse 2022), suggesting that anaerobic planktonic microorganisms are responsible for their production, in line with the presence of brGMGT‐1a (*m/z* 1020) in the euxinic waters of the Black Sea.

**FIGURE 6:**
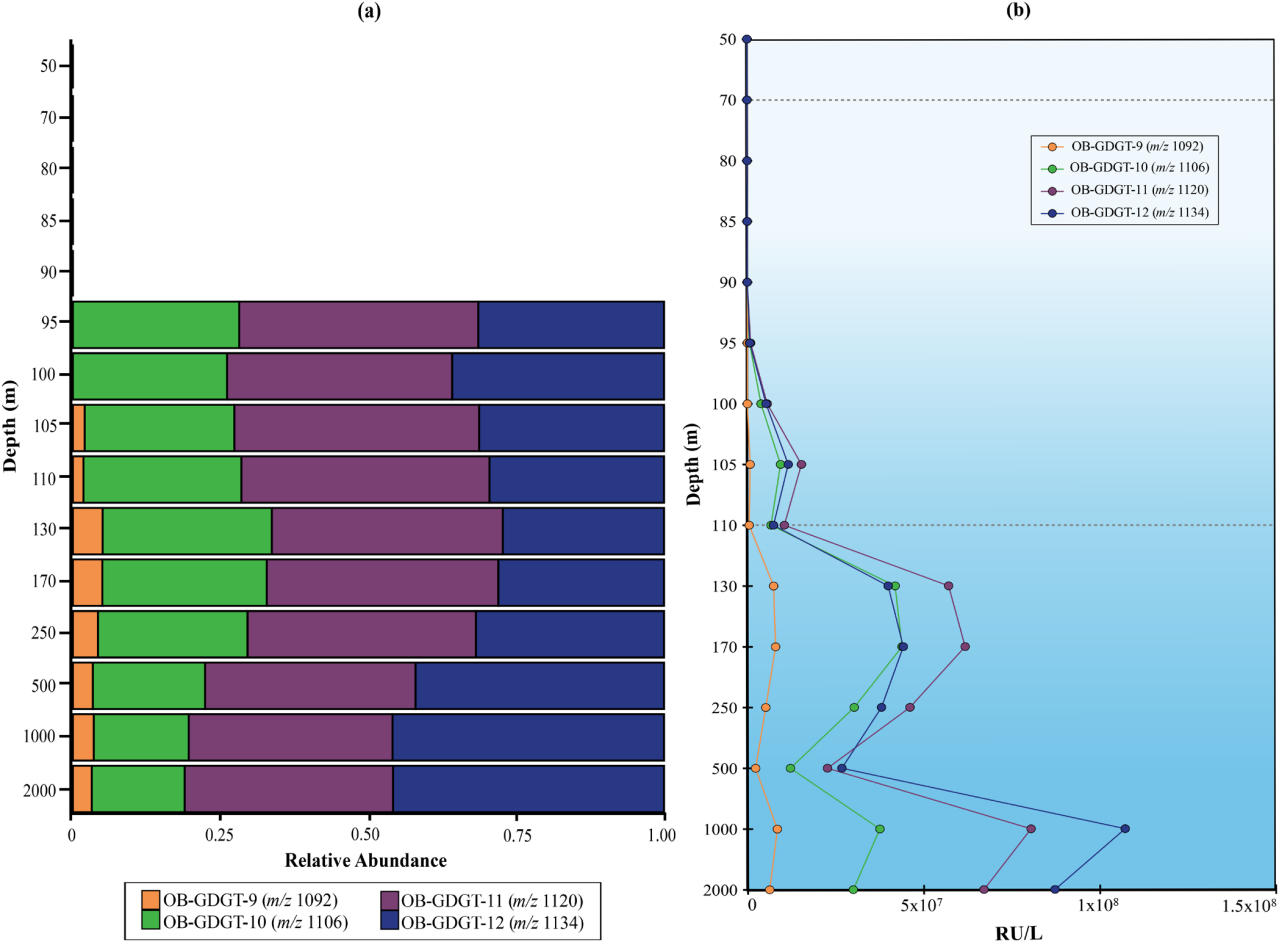
Distribution of overly branched OB‐GDGTs in the Black Sea water column, (a) relative abundance, (b) response units per litre. Dotted lines indicate the beginning of suboxic (70 m) and euxinic (110 m) zones.

We also detected a diversity of OB‐GDGTs: OB‐GDGT‐9 (*m/z* 1092), OB‐GDGT‐10 (*m/z* 1106), OB‐GDGT‐11 (*m/z* 1120) and OB‐GDGT‐12 (*m/z* 1134) (see Figures 6A, S4F‐I, Table S6), which had been previously detected in the Black Sea water column by Liu et al. (2014). The distribution of the OB‐GDGTs is similar to those detected by Liu et al. (2014), with the only exception, that the concentration of the regular brGDGTs and OB‐GDGTs in our dataset decreases at 2000 m, while Liu et al. (2014) report a consistent increase with depth (Figure 6B), as also seen for the brGDGTs.

Zeng et al. (2023) investigated OB‐GDGTs in sediments and observed a higher degree of methylation in the OB‐GDGTs with increasing depth higher % of OB‐GDGT‐9 (*m/z* 1092), OB‐GDGT‐10 (*m/z* 1106), and OB‐GDGT‐12 (*m/z* 1134) in deeper sediment depths. A similar observation was encountered in the marine water columns of the Black Sea and the Cariaco Basin (Liu et al. 2014).

Nevertheless, in our study, the detected OB‐GDGTs did not change in relative abundance compared to each other with depth, although a slight decrease in OB‐GDGT‐10 and an increase of OB‐GDGT‐12 has been observed (Figure 6a,b) We suggest that the production of branched and OB‐GDGTs in the deep waters of the Black Sea is not consistent between different sampling sites and seasons, and may be associated with different producers or differences in the abundance of the existing populations at the deeper Black Sea waters.

Something to note is that the profile of both brGDGTs and OB‐GDGTs (Figures 5B and 6B) is comparable to the profile of total archaeal and bacterial 16S rRNA gene reads (Figure S3). This is especially notable in the increase in relative abundance at 1000 m for both lipid and gene profiles, and the evident decrease at 500 and 2000 m. The only depth where the profiles do not align is at 130 m where the peak in relative lipid abundance of GDGTs does not correspond to a peak in gene copies. Regarding the potential sources of brGDGTs and OB‐GDGTs in marine anoxic systems, Zeng et al. (2023) performed a co‐occurrence network analysis correlating the relative abundance of those lipids with that of the bacterial communities based on 16S rRNA gene amplicon sequencing. They suggested that members of the bacteria Chloroflexi, Proteobacteria and Dadabacteria could be the potential producers of brGDGTs and that Armatimonadota, Planctomycetota and Chloroflexi could be the producers of OB‐GDGTs in the Mariana Trench sediments, pending further confirmation.

#### 2.2.3 Inference of iso‐ and brGDGTs Producers Based on Their Lipid Biosynthetic Genomic Potential

As mentioned above, previous studies have inferred the biological sources of iso‐, br‐ and OB‐GDGTs by correlating the presence of lipids in environmental samples with the occurrence of archaeal and bacterial groups based on 16S rRNA gene amplicon sequencing (Buckles et al. 2013; Sollai et al. 2019; Baxter et al. 2021; De Jonge et al. 2021; Zeng et al. 2023). However, this inference does not necessarily correctly link a product (lipid) with its sources as correlation does not imply causation (Ding et al. 2024). To make a more accurate link between lipid and its producer(s), here we targeted the genomic capacity to synthesise these lipids in the assemblies of the sequenced metagenomes of the 15 samples throughout the Black Sea water column (~1.64 × 10^8^ scaffolds and 5844 metagenome‐derived genomes (MAGs)) (see Section 4 for details), and compared the distribution of the hits with the distribution of the observed membrane lipids.

**FIGURE 7:**
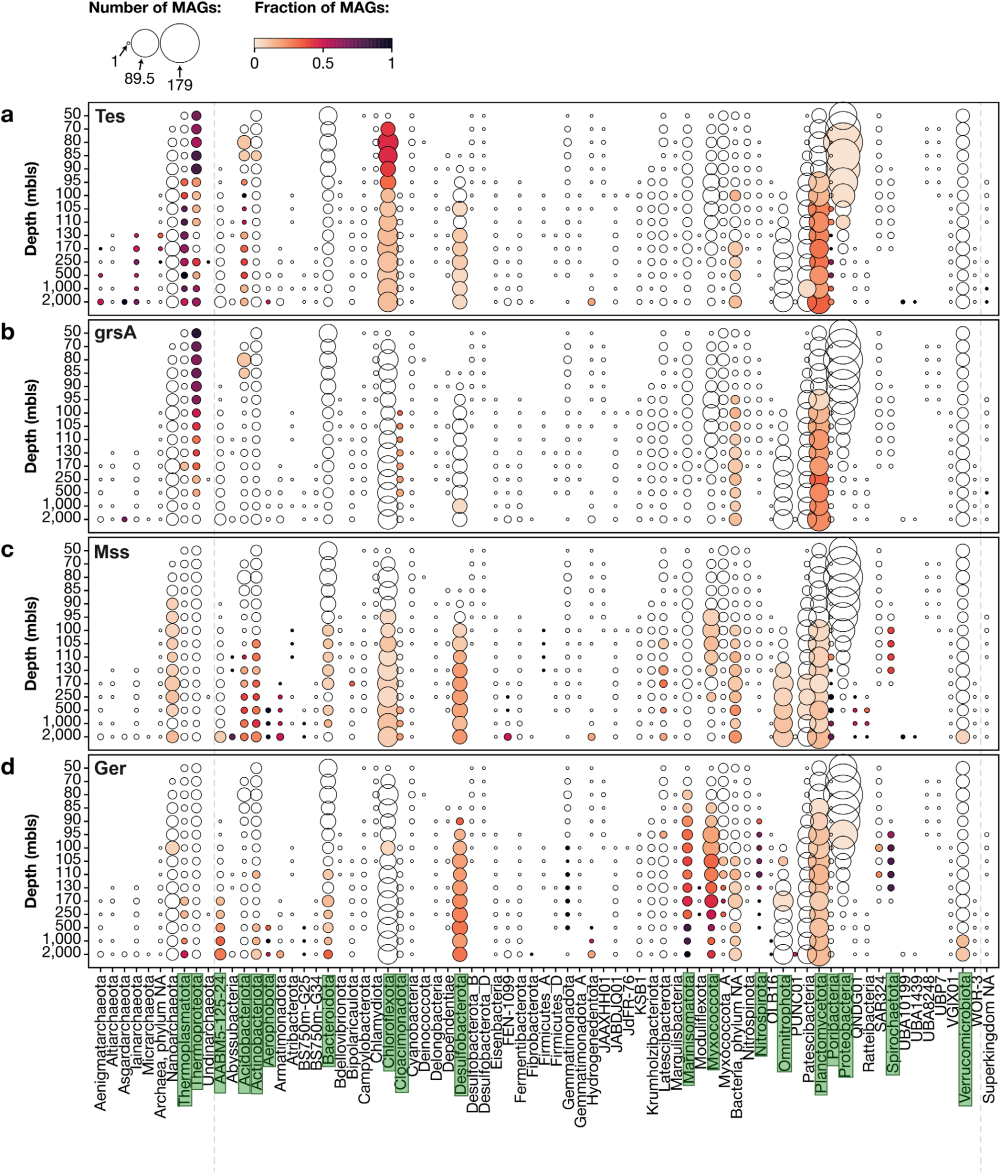
Number of metagenome‐assembled genomes (MAGs) binned in the Black Sea, grouped by sample (depth) and taxonomy (rank: Phylum). Circle size represents the number of MAGs by sample/taxonomy, colour intensity describes the fraction of MAGs containing protein homologue: (a) Tes; (b) GrsA; (c) Mss; (d) Ger.

**FIGURE 8:**
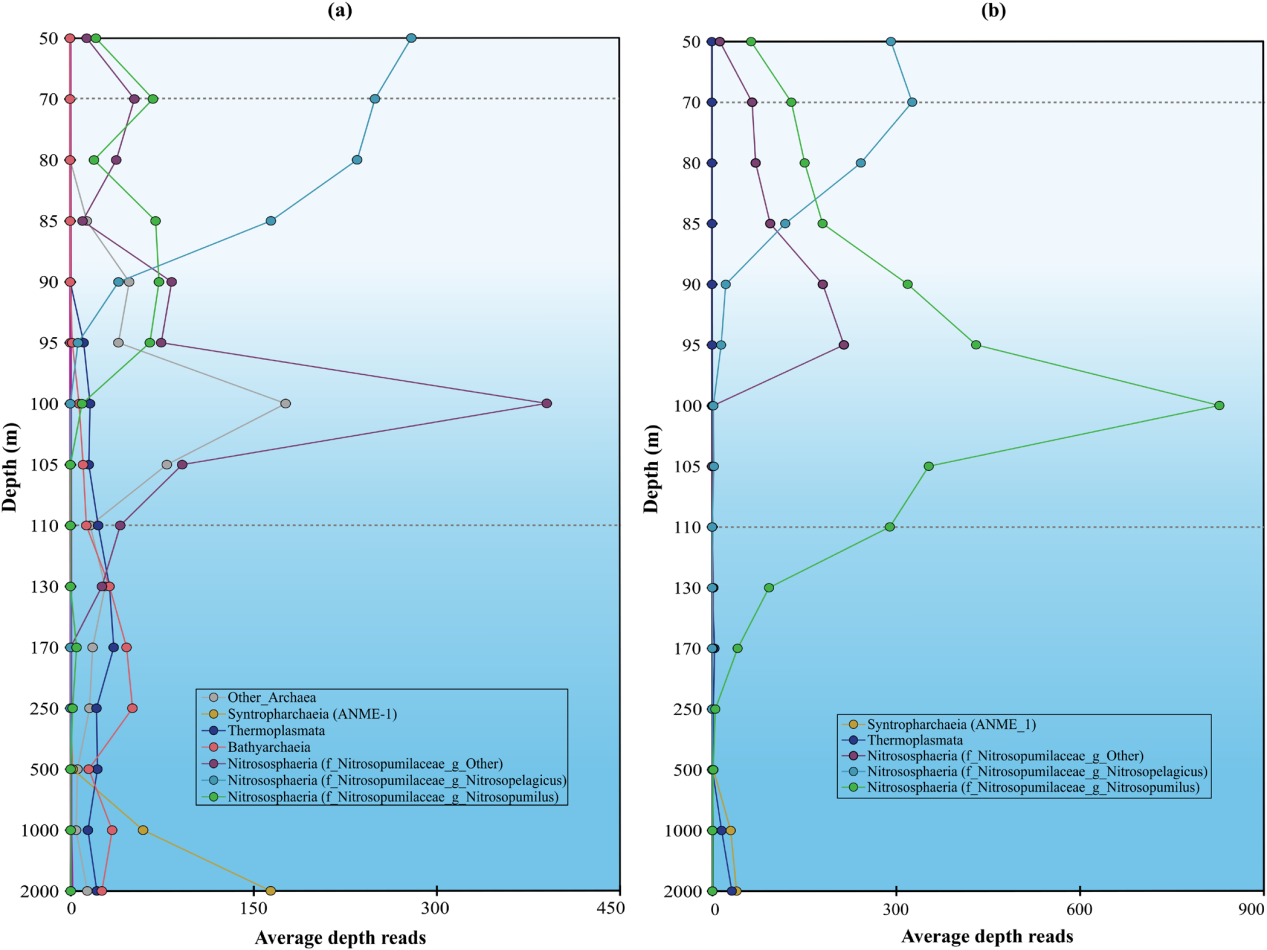
The sum of the average depth (i.e., number of mapped reads per base pair, per 1e+8 mapped reads) of Tes protein homologue hits (a) and of GrsA protein homologue hits (b) (see Experimental procedures for details) detected in archaeal groups across the Black Sea SPM profile from 50 to 2000 m depth. (taxa marked with * have been plotted on the secondary axis for better visualisation). Dotted lines indicate the beginning of suboxic (70 m) and euxinic (110 m) zones.

**FIGURE 9:**
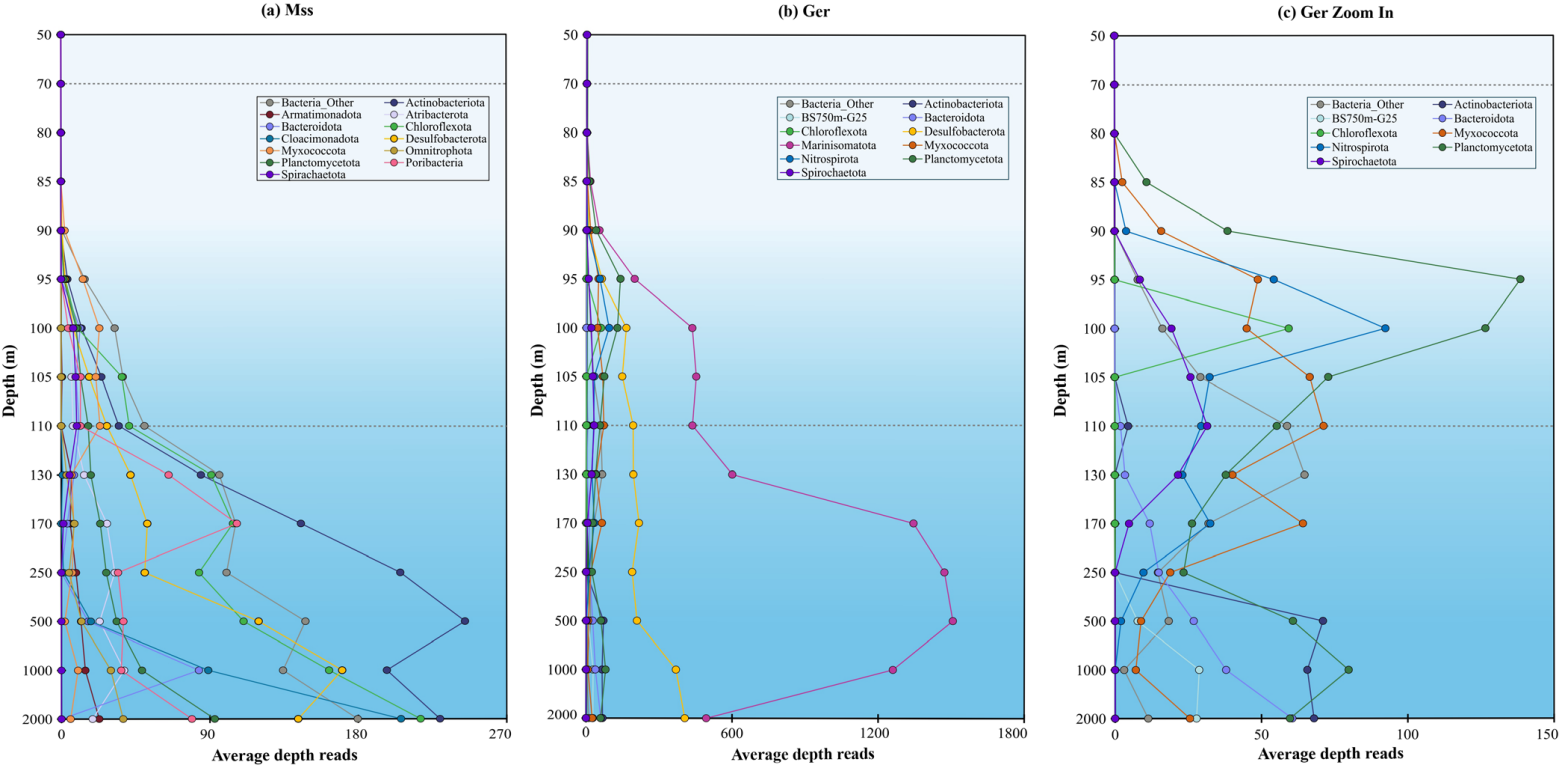
The sum of the average depth (i.e., number of mapped reads per base pair, per 1e+8 mapped reads) of the Mss protein homologue hits (a) and of the Ger protein homologue hits (b) with detail of less abundant taxa (c) (see Section 4 for details) detected in bacterial groups across the Black Sea SPM profile from 50 to 2000 m depth. Dotted lines indicate the beginning of suboxic (70 m) and euxinic (110 m) zones.

We performed two complementary analyses. First, we examined the fraction of MAGs from a certain phylum in each sample that encode a relevant key gene to synthesise the lipid of interest, which reflects the within‐phylum strain diversity and the universality of the biosynthetic capacity in the phylum at different depths in the water column (Figure 7, S10). This shows niche‐based differentiation of different strains within a phylum, for example when all MAGs of a phylum in the upper water column have the biosynthetic capacity to synthesise a lipid and few in the deep waters do. In addition, this analysis allows for an assessment of noise due to technical artefacts, as it is likely that a high fraction of MAGs encoding a gene points to its true presence within the phylum instead of a false positive due to spuriously binned genes, especially if they represent a large number of MAGs (large circles and dark colours in Figure 7, S10). Second, we quantified the abundance of hits in the assembly irrespective of whether the scaffold was binned or not, using normalised depth based on read mapping to the scaffolds (i.e., number of mapped reads per base pair per 1 × 10^8^ mapped reads, see Section 4 for details), which allows for a comparison of sequenced DNA between samples (Figures 8, 9, S5–S9). This second analysis takes abundance into account and allows for a correlation with the lipid profile. Moreover, since also unbinned scaffolds are examined, it represents a more comprehensive picture of the microbiome than the MAGs alone (Hauptfeld et al. 2024), even if the taxonomic annotations of scaffolds from highly unknown microorganisms are less robust than those of MAGs (Meijenfeldt et al. 2019). We briefly summarised the findings of this study in Table S16, where based on the analysis of this paper, all the major taxa associated with the genes of interest based on metagenomic annotation are correlated with the potential lipids they synthesise.

For the case of the genomic capacity to synthesise isoGDGTs, here we determined the distribution and abundance of the detected homologues (see Experimental procedures for details) of Tes (GDGT synthase; Zeng et al. 2022) involved in the coupling of isoprenoids leading to GDGT without and with cyclopentane rings, and of the gyrase GrsA involved in the GDGT cyclization up to 4 rings (Zeng et al. 2019). Tes gene hits were detected in different microbial groups throughout the water column, within Archaea mostly in the phylum Thermoproteota (classes Nitrosphaeria and Bathyarchaeaia) and class Thermoplasmata (Figures 7A, 8A, Tables S8, S9AB). The distribution of isoGDGTs (Figures 3B, 4) can be compared to the abundance profile of Tes hits (Figure 8A), with those of the Thaumarchaeota genus *Nitrosopelagicus* more abundant in the surface waters (i.e., 70 m), followed by a maximum of Tes hits attributed to the Thaumarchaeota genus *Nitrosopumilus* in the upper suboxic waters (i.e., 100 m), while Tes hits of Bathyarchaeia and Thermoplasmata were more abundant in the upper euxinic waters (250 m), followed by an increase with depth of Tes hits of members of the ANME‐1 Archaea (class Syntropharchaeia) (Figure 8A, Tables S8, S9AB).

This profile coincides with the distribution of hits of the gyrase GrsA for Thaumarchaeota (equivalent to GTDB‐Tk classification Thermoproteota) genera *Nitrosopelagicus* and *Nitrosopumilus* (Figure 8B, Tables S8, S10AB), indicating that those two genera are likely the main and only producers of isoGDGTs with rings in the surface and suboxic waters. In the euxinic waters, we detected GrsA hits in low abundance attributed to ANME‐1 (class Syntropharchaeia; 3 unbinned scaffolds from the 3 deepest samples), as well as to class Thermoplasmata 3 unbinned scaffolds from 250 m depth, 1000 m depth, and 2000 m depth, and 1 binned scaffold from 170 m depth (Figure 8B, Table S10AB), suggesting these two archaeal groups could be responsible for the biosynthesis of isoGDGTs with rings in the deep waters of the Black Sea. However, evidence supporting this is weak as the scaffolds where those GrsA homologues were detected are unbinned and their abundance is low. Is worth noting that members of the Thermoplasmata have been widely detected in marine sediments and anoxic water columns often co‐occurring with ANME‐1 (Blumenberg et al. 2004; Suominen et al. 2021; Iasakov et al. 2022; Wegener et al. 2022). If members of the Thermoplasmata present in similar settings would harbour the capacity to make isoGDGT‐1 and 2, it would compromise the use of these isoGDGTs as markers for anaerobic methane oxidation by Archaea.

Tes homologues were also detected in 281 bacterial MAGs (Figure 7A, Table S8). The presence of homologues of this gene in bacterial genomes was also observed in a microbial genome screening by Zeng et al. (2022) and could be indicative of their potential to synthesise brGDGTs. Although the presence of Tes homologues in bacterial genomes has not been experimentally confirmed to provide the capacity to synthesise bacterial membrane‐spanning lipids, here we report the distribution of Tes hits in bacterial genomes (Table S9AB), with members of the phylum Chloroflexota more abundant in the surface and upper suboxic waters, while others were more abundant in the lower suboxic and upper euxinic waters such as phyla Acidobacteriota, Desulfobacterota, Poribacteria and Planctomycetota, with the latter dominant in the suboxic and euxinic waters with a maximum at 2000 m depth (Figure S5A). The bacterial diversity of Tes homologues detected in the MAGs (Figure 7A) includes comparable taxonomic groups, with the most diverse groups being phyla Chloroflexota (Tes homologues detected in 111 out of 684 MAGs) and Planctomycetota (110 out of 684 MAGs), followed by Acidobacteriota (17 out of 97 MAGs), Desulfobacterota (10 out of 262 MAGs) and Poribacteria (10 out of 29 MAGs) (Table S8).

Hits of the gyrase GrsA enzyme were mostly detected in bacterial MAGs of phyla Planctomycetota (94 out of 684 MAGs) and Cloacimonadota (7 out of 42 MAGs) and were noticeably missing from members of the phylum Chloroflexota (Figure 7B, S5B, Tables S8, S10AB). Some additional hits were detected in Acidobacteria and Desulfobacteriota (Figure S5B). We did not detect any hits of Tes (in MAGs and unbinned scaffolds) in phylum Cloacimonadota, possibly suggesting that the GrsA homologues in the phylum have a different function than GDGT cyclization or that Cloacimonadota encode a non‐homologous gene that replaces the putative Tes‐like functionality (note that many of the Cloacimonadota MAGs also do not encode Mss, see in Figure 7C). Although the functional roles of Tes and GrsA have not been assessed in bacterial genomes, members of the Planctomycetota could potentially synthesise some of the brGDGTs with rings reported in our study (Figure 5).

In addition, Black Sea MAGs were screened for the presence of proteins involved in the confirmed synthesis of bacterial membrane‐spanning (ether) lipids, namely Mss and Ger enzymes involved in the coupling of fatty acids and of the ether bond formation, respectively (Sahonero‐Canavesi et al. 2022). Mss hits (Tables S11AB) were abundant and detected in both unbinned scaffolds and MAGs of several bacterial phyla (Table S8). The abundance profile based on unbinned scaffolds (Figure 9A), shows phylum Actinobacteriota as the most abundant microbial group, which increased with depth from 110 m downwards with maximum abundances at 500 m; phyla Chloroflexota, Cloacimonadota and Planctomycetota with a maximum abundance at 2000 m; and phyla Desulfobacterota and Bacteroidota with a maximum abundance at 1000 m (Figure 9A). The MAGs diversity profile (Figure 7C) includes the same taxonomic groups: Actinobacteriota (Mss homologues detected in 17 out of 146 MAGs), Chloroflexota (25 / 558 MAGs), Cloacimonadota (3 / 42 MAGs) and Planctomycetota (27 / 684 MAGs), Desulfobacterota (34 / 262 MAGs) and Bacteroidota (7 / 273 MAGs) with Mss protein‐coding sequences additionally detected in MAGs of the phyla Acidobacteria (11 / 97 MAGs), Armatimonadota (7 / 13 MAGs), Latescibacteriota (7 / 92 MAGs), Myxococcota (12 / 347 MAGs), Omnitrophota (15 / 267 MAGs), Patescibacteria (3 / 428 MAGs), Poribacteria (14 / 29 MAGs), Spirochaeota (8 / 28 MAGs), amongst others (Figure 7C, Table S8). This diverse group of bacterial phyla hints at a more widespread potential to synthesise membrane‐spanning lipids in marine bacterial communities than previously considered. Although we did not detect any Tes hits in Cloacimonadota, the presence of hits for both GrsA (7/42 MAGs) and Mss (3/42 MAGs) could hint at the potential of this group to produce brGDGTs with rings, although co‐occurrence of both genes in the same genome is inconclusive. Only 1 of the 42 surveyed MAGs has both enzymes, and this could be explained by either contamination, incomplete assembly and/or binning, or due to specific environmental conditions under which some of the organisms of this phylum could produce MSL using both enzymes.

Homologues of Ger (glycerol ester reductase) were detected in a diverse group of bacteria. The phylum Marinisomatota was amongst the most abundant in suboxic and euxinic waters (Figure 9B). Based on the unbinned scaffolds (Table S12AB), order UBA8477 was distributed towards the upper waters and the order Marinisomatales had a maximum abundance at 500 m. Marinisomatota was also amongst the most diverse groups (Ger homologues detected in 45 out of 134 MAGs) (Figure 7D, Table S8). Other abundant (Figure 9C) and diverse (Figure 7D, Table S8) phyla were the Myxococcota (61/347 MAGs), Desulfobacterota (54/262 MAGs), Planctomycetota (53/684 MAGs), Spirochaeota (19/28 MAGs), Nitrospirota (14/23 MAGs), Bacteroidota (9/273 MAGs), Actinobacteriota (4/146 MAGs) and Aerophobota (4/7MAGs) (Tables S8, S12AB).

Genomic coincidence of Mss and Ger hits were found in 34 MAGs of Desulfobacterota, 27 MAGs of Planctomycetota, 12 MAGs of Myxococcota and 4 MAGs of Actinobacteria, pointing to their potential genomic capacity to synthesise brGDGTs and suggesting these might be the bacterial groups responsible for producing brGDGTs and OB‐GDGTs detected in the Black Sea water column in this study. Note that Ger is a close homologue of PlsA, which is an enzyme involved in the production of alkenyl ethers (i.e., plasmalogens). It is currently not possible to discriminate if the presence of these homologues leads to the formation of alkyl or alkenyl ethers based on sequence homology only (Sahonero‐Canavesi et al. 2022), and the distribution of Ger‐like hits in different bacterial MAGs can also indicate the potential to produce plasmalogens in some of these groups, which were not part of this study.

Homologues of genes encoding the ElbD and Agps proteins of *M. xanthus* were also screened in the metagenomes to constrain the presence and abundance across the water column in different taxonomic groups which might harbour the capacity to synthesise alkyl or alkenyl ether bonds, respectively. For the case of ElbD, hits were found in a diverse group of bacterial phyla (Figure S10A Table S8). The most abundant (Figure S6, Table S13AB) groups in the water column were the phyla Myxococcota, distributed predominantly in the upper oxic and suboxic waters; Chloroflexota abundant in upper suboxic and euxinic waters (peaks at 85, 500 and 2000 m); Proteobacteria in the upper suboxic waters; Actinobacteria, Omnitrophota and the candidate division AABM5‐125‐24 of the FCB superphylum in the euxinic waters (Figure S6). These phyla were also amongst the most diverse (Figure S10A Table S8): Myxococcota (121/347 MAGs), Omnitrophota (93/267 MAGs), division AABM5‐125‐24 (58/69 MAGs), Proteobacteria (45/932 MAGs), Chloroflexota (37/558 MAGs); although other phyla containing many MAGs that encode elbD homologues (Figure S10A) were found: Latescibacterota (50/92 MAGs), Verrucomicrobia (43/270 MAGs) and Planctomycetota (33/267 MAGs). Homologues to the gene encoding the Agps protein involved in the formation of alkenyl ethers in *Myxococcus* were also found in a wide diversity of bacterial MAGs binned from the Black Sea water column metagenomes (Figure S10B, Tables S8, S14). Hits with the highest abundance across the water column were affiliated with phyla Actinobacteria, Proteobacteria and Verrucomicrobiota in the suboxic waters and with phyla Marinisomatota, Desulfobacterota and Chloroflexota in euxinic waters (Figure S7). These phyla also corresponded to the most diverse: Desulfobacterota (Agps homologues detected in 59/262 MAGs), Chloroflexota (56/558 MAGs), Marinisomatota (49/134 MAGs), Proteobacteria (42/932 MAGs) and Verrucomicrobiota (43/270 MAGs) (Figure S10B). Although not very abundant, Myxococcota (120/347 MAGs), Latescibacterota (67/92 MAGs) and Myxococota were amongst the most diverse phyla encoding *agps* homologues (Figure S10B).

Our metagenomic screening supports that the potential genomic capacity to synthesise alkyl ethers via Ger or ElbD, and alkenyl ethers via Agps is harboured by a wide diversity of bacterial groups which seem to be distributed in different niches throughout the Black Sea water column. MAGs of the phylum Verrucomicrobia, specifically of the *Pontiella* genus, were found to contain homologues coding for the ElbD protein. Members of the *Pontiella* genus have been isolated in a previous study from Black Sea samples and their lipids have been analysed, but no ether‐bound lipids were detected (Vliet et al. 2020). This emphasises that the genomic presence of homologues of this gene does not necessarily imply the synthesis of the lipids, either because the gene is not involved in lipid biosynthesis or because it is not expressed under laboratory conditions. MAGs affiliated with Acidobacteria containing *elbD* homologues (15/97 MAGs) were also found in the Black Sea water column present throughout suboxic and euxinic waters (Tables S8, S13AB). Some members of the Acidobacteria are known to synthesise brGDGTs (Sinninghe Damsté et al. 2014, 2018), but the pathway used for their biosynthesis is still unclear as only some genomes of Acidobacteria subdivision 4 harbours the *elb* gene cluster or *agps* homologues and the role of the proteins that they encode in the ether bond formation has not been conclusively demonstrated. Therefore, it is unclear if the uncultured Acidobacteria detected in the Black Sea could be potential sources of the detected brGDGTs; however, it is worth mentioning that 9 MAGs of the phylum Actinobacteriota (class Humimicrobiia) obtained from our dataset have been seen to harbour homologues of both the Mss (Tables S8, S11AB) and of the ElbD proteins (Tables S8, S13AB), indicating genomic potential of members of this group to synthesise brGDGTs using the *elb* gene cluster to make alkyl ether bonds rather than by using the Ger protein. In this regard, out of the 558 MAGs from the phylum Chloroflexota present in the Black Sea water column, only 25 MAGs were detected with Mss and 37 MAGs with ElbD, and from these, a small fraction (9 MAGs) contains homologues of both protein‐coding genes, suggesting different potential genomic capacities to make brGDGTs within the same phylum (Tables S11AB–S13AB). The same applies to MAGs of members of the phylum Myxococcota (12 MAGs with Ms.'s homologues, 61 MAGs with Ger homologues, 121 MAGs with ElbD homologues, 120 MAGs with Agps homologues, and a smaller subset of MAGs with two or more of the homologues).

The phylum Planctomycetota represents an even stranger case. Homologues of Tes and GrsA (previously thought to be exclusive to Archaea) were detected in some representatives of the class Phycisphaerae of this phylum, while Mss and Ger homologues were detected in the Brocadiia, Phycisphaerae and Planctomycetia classes. This suggests that members of the Planctomycetota phylum can be a source of very diverse lipids, including archaeal‐like lipids with rings or brGDGTs. Some members of Brocadiia have been seen to produce alkyl ethers (Rattray et al. 2008), which in some cases coincides with the presence of either Ger, ElbD or Agps homologues in their genome (Sahonero‐Canavesi et al. 2022), thus it is possible that this group performs the biosynthesis of ether lipids utilising alternative biochemical strategies.

Recently, a radical SAM protein, a glycerol monoalkyl glycerol tetraether (GMGT) synthase (Gms), has been confirmed to be responsible for cross‐linking the two hydrocarbon tails of isoprenoidal GDGT to produce isoprenoidal GMGTs (Garcia et al. 2024). The study by Garcia et al. 2024 also detected the presence of Gms homologues in bacterial genomes of the Acidobacteria, Planctomycetes, and diverse bacterial candidate phyla, and suggested that those homologues could be involved in the formation of the H covalent bond also in bacterial brGDGTs to generate brGMGTs. To this end and considering the detection of brGMGT‐1a (*m/z* 1020) in our dataset, we also screened for the presence of homologues of the Gms protein in the Black Sea metagenomes. Most of the detected Gms homologues were affiliated with the phylum Desulfobacterota (84/262 MAGs) with maxima in the upper suboxic and deep euxinic waters, and with different groups of the Planctomycetota (90/684 MAGs) with higher abundance in the deeper suboxic (130–170 m) and euxinic waters (2000 m) (Figures S9, S10C Tables S8, S15AB). The abundance of Gms homologues from Planctomycetota coincides with the distribution of brGMGT‐1a (*m/z* 1020) (Figure 5a,b) with maxima at around 130 m, suggesting these Planctomycetota groups might be responsible for the synthesis of these lipids.

Surprisingly, hits of Mss, Ger, ElbD and Agps were also detected in archaeal MAGs. Mss hits were only detected in the class Woesearchaeia (DPANN superphylum) with maximum abundance from 100 to 130 m depth (Figure 7C, S19A), which also partially coincided with the distribution of the Ger and ElbD hits found in some DPANN Woesearchaeia MAGs (Figure S9BC). Although it has not been experimentally confirmed whether different taxa within Woesearchaeaia are free‐living or symbionts of an archaeal or bacterial host, it has recently been found that some Woesearchaeota MAGs carried genes for cyclopropane fatty acid phospholipid synthesis, which are used in stabilising bacterial phospholipid membranes (Zhang et al. 2024). This combined evidence might suggest that members of this DPANN group could potentially be synthesising or modifying membrane lipids to make bacterial membrane‐spanning ether‐based lipids in the suboxic zone. Although hypothetical, this finding could further support the potential for DPANN archaea to synthesise bacterial‐like lipids (Castelle et al. 2021; Villanueva et al. 2021), likely coming from a bacterial host as they do not have the biosynthetic genetic capacity to make the building blocks themselves (Castelle et al. 2018; Dombrowski et al. 2019, 2020).

## 3 Conclusions

By using a combination of metagenomics and targeted lipid analysis by high‐resolution accurate mass spectrometry in environmental samples, we have been able to better connect specific membrane lipids with their biological sources. We were able to identify previously unreported taxa in the Black Sea (i.e., Altiarchaea and Aenignmarchaea), as well as a high diversity of brGDGTs and OB‐GDGTs, including GMGTs lipids.

In the euxinic waters, archaea of the Thermoplasmatota may contribute to the production of isoGDGT with rings while previously members of ANME‐1 were expected to be the only source. This might affect the use of isoGDGTs with 1 or 2 rings as biomarkers of anaerobic methane oxidation. Furthermore, we also suggest Planctomycetota, Cloacimonadota, Desulfobacterota, Chloroflexota, Actinobacteria and Myxococcota as potential producers of brGDGTs and/or OB‐GDGTs based on the detection of MSL and ether‐forming coding genes in their MAGs. Members of these groups would make ideal candidates for cultivation attempts and physiological experiments to further investigate their membrane lipid composition. There are major cultivation challenges to overcome to isolate high‐pressure anaerobic microorganisms to directly study their lipids, but several successful attempts have been published in recent years (Pappenreiter et al. 2019; Quéméneur et al. 2019; Vliet et al. 2020; Yadav et al. 2021). By applying reverse metagenomics and understanding the metabolic potential of these taxonomic groups, it is also possible to design novel cultivation strategies for groups of interest, like Thermoplasmatota or Bathyarchaeaia (Cross et al. 2019; Sun et al. 2020; Lewis et al. 2021).

Based on the detection of homologues of the archaeal *tes* and *grsA* genes, we also suggest bacteria of the Planctomycetota and Cloacimonadota could be synthesising membrane‐spanning lipids potentially with cyclopentane rings. Confirming this hypothesis would be essential to elucidate how the ‘lipid divide’ occurred (Koga 2011; Lombard, López‐García, and Moreira 2012; Villanueva, Schouten, and Damsté 2017; Villanueva et al. 2021; Sahonero‐Canavesi et al. 2022), and if the physiological advantage of harbouring membranes with mixed archaeal and bacterial features is to increase resilience and adaptability to environmental stress.

We also detected homologue of the bacterial MSL and ether‐forming coding genes in Archaea, specifically in members of the DPANN Woesearchaea. Because of the restricted genomic potential, this group possesses to produce their own lipids (Castelle et al. 2018; Dombrowski et al. 2019, 2020) this might suggest their ability to modify already formed membrane lipids taken from a bacterial host and the capability to make bacterial membrane‐spanning ether‐lipids. Identifying the prospective hosts of Woesearchaea in euxinic waters and the isolations of host‐symbiont pairs could lead to a better understanding of the physiological and evolutionary dynamics of these archaea.

## 4 Experimental Procedures

### 4.1 Samples

Suspended particulate matter (SPM) from 15 depths across the water column (50–2000 m) was collected at sampling station 2 (N42°53.8′, E30°40.7′, 2107 m depth) during the Phoxy cruise 64PE371 (BS2013) on 9–10 June 2013 as previously described (Villanueva et al. 2021). SPM was collected with McLane WTS‐LV in situ pumps (McLane Laboratories Inc., Falmouth) on pre‐combusted glass fibre filters with 142‐mm diameter and 0.7‐μm pore size.

### 4.2 Lipid Analysis

Total lipids were extracted from freeze‐dried glass fibre filters as described in Sollai et al. (2019). The Bligh and Dyer lipid extracts were analysed for archaeal GDGTs intact polar lipids (IPLs), which are composed of the core lipid (CL) attached to one or two polar head groups, by Ultra High‐Pressure Liquid Chromatography (UHPLC) system (an Agilent 1290 Infinity I) coupled to a Q Exactive Orbitrap high‐resolution mass spectrometry (HRMS) system (Thermo Fisher Scientific, Waltham, MA). UHPLC–atmospheric pressure chemical ionisation MS for archaeol and isoGDGTs, was done according to Hopmans, Schouten, and Sinninghe Damsté (2016) with some modifications as described in Sollai et al. (2019). SPM samples were re‐extracted also following the Bligh and Dyer lipid extraction procedure but with extractions of the residue with a mixture of methanol, dichloromethane, and aqueous trichloroacetic acid solution (TCA) pH 3 (2:1:0.8, v:v) as described in Bale et al. (2021). These extracts were analysed for brGDGTs and OB‐GDGTs core lipids by using UHPLC‐HRMS according to the reversed phase method of Wörmer et al. (2013) with the modifications by Bale et al. (2021).

The abundance of brGDGTs was based on their individual MS peak area response. As different lipids may have different response behaviour, the relative abundance of peak area does not necessarily reflect the actual relative abundance of the different lipids. However, this method allows for comparison between the samples analysed in this study. The peak areas were determined from extracted ion chromatograms of the combined [M + H]^+^, [M + NH_4_]^+^ and [M + Na]^+^ ion (where present) for each individual lipid species. These peak areas were normalised based on the response of an internal standard, a deuterated betaine lipid (1,2‐dipalmitoyl‐sn‐glycero‐3‐O‐4′‐[*N*,*N*,*N*‐trimethyl(d9)]‐homoserine; Avanti Lipids). This allowed us to account for matrix effects and variations in mass spectrometer performance, and to correct reported peak areas for these effects.

A range of branched GDGTs (brGDGTs) were detected (Figures S1, S4 for structures and nomenclature). Figure S4 shows the distribution of brGDGTs in SPM from 1000 m depth. The identification of all the brGDGT components was based on their accurate masses (Table S7) and by comparison with published fragmentation spectra (Liu, Summons, et al. 2012; Naafs et al. 2018, 20; Baxter et al. 2019). Further confirmation of the assignments, and particularly their elution order, came from a re‐examination of the brGDGT distribution in extracts from Lake Challa, Kenya (data not shown), under both normal phase analysis (Baxter et al. 2019) and reverse phase analysis (Mitrović et al. 2023). This allowed us to confirm the elution of brGMGT‐1a (a ‘H‐GDGT’, also known as brGDGT H1020) relative to brGDGT‐1a. Indeed, the elution of brGMGT‐1a during this study, under reverse phase analysis, was somewhat unexpected, forming a double peak (presumably two isomers) with maxima eluting around 0.4 and 0.7 min after that of brGDGT‐1a (*m/z* 1022). Under normal phase analysis (e.g., Baxter et al. 2019) brGMGTs elute 20 min after their equivalent brGDGTs. Furthermore, during reverse‐phase analysis, isoprenoidal GMGTs elute a few minutes before their equivalent GDGTs (Mitrović et al. 2023). Several late eluting, overly branched (OB)‐GDGTs (Liu, Summons, et al. 2012) were also detected (Figure S4; Table S7), identified from their accurate masses and their elution pattern.

### 4.3 DNA Extraction, 16S rRNA Gene Amplicon Sequencing and Quantitative PCR

The DNA extraction from SPMs across the water column of the Black Sea was reported in Sollai et al. (2019). Microbial diversity determination by 16S rRNA gene amplicon sequencing was performed as described in (Villanueva et al. 2021). Quantitative PCR was performed using the same primers of the 16S rRNA gene amplicon sequencing as described in Villanueva et al. (2021).

### 4.4 Metagenomic Sequencing, Assembly and Binning

The metagenomic sequencing, assembly, and binning are described and reported in Ding and von Meijenfeldt et al. (2004). In short, the 15 samples were individually assembled, sequencing reads were mapped back to the assembled scaffolds generating 15 × 15 mappings, and the scaffolds were binned per sample into 5844 metagenome‐assembled genomes (MAGs). MAG quality was assessed with CheckM v1.1.3105 (Parks et al. 2015) in the lineage‐specific workflow, and the MAGs were taxonomically annotated with GTDB‐Tk v2.1.0 (Chaumeil et al. 2022) using release 207 of the GTDB (Parks et al. 2022). The GTDB‐Tk annotations were only used for the reconstruction of the archaeal phylogeny (see below) and in Figure 2A and Table S4. Taxonomy was also assigned to scaffolds and MAGs with Contig Annotation Tool (CAT) and Bin Annotation Tool (BAT) (Meijenfeldt et al. 2019), respectively, from the CAT pack software suite v5.2.3, using a reference database based on the set of non‐redundant proteins in GTDB release 207. The CAT and BAT classifications were used in all other analyses. Prodigal v2.6.3 in metagenomic mode (Hyatt et al. 2012) and DIAMOND v2.0.6109 (Buchfink, Reuter, and Drost 2021) were used for protein prediction and alignment, respectively. The ‐top parameter was set to 11 for CAT and to 6 for BAT. A MAG may get multiple taxonomic annotations with the default parameter settings of BAT (−f 0.3) in which case we took the majority classification (−f 0.5).

### 4.5 Archaeal Phylogeny

A phylogeny was constructed representing the archaeal MAGs detected in the Black Sea and a set of reference genomes picked from across the archaeal tree of life with a high taxonomic sampling around the taxonomic groups that were detected in the Black Sea. The taxonomy of archaeal MAGs was assessed based on the GTDB‐Tk annotations (see above). Similar MAGs in the Black Sea were identified with dRep v3.4.0 (Olm et al. 2017) using the fastANI algorithm (‐‐P_ani 0.9, ‐‐S_ani 0.99) and only including MAGs with a minimum completeness of 50% (‐‐completeness 50). We downloaded representative genomes from GTDB, selecting one archaeal genome per order, and all representative genomes of clades that contain the archaeal MAGs from the Black Sea. The representative genome that was chosen per order was selected based on estimated CheckM completeness—5 × contamination. Genes were identified with Prodigal v2.6.3 in single mode (Hyatt et al. 2010), and queried for the 27 Clusters of Orthologous Gene (COG) families that showed evidence of being primarily vertically transferred (Moody et al. 2022), as described in (Gallego et al. 2024). Overall, 24 COG families were selected, and genes from these families that were present in single‐copy in a genome were extracted and aligned with MAFT v7.505 (Katoh and Standley 2013) using the L‐INS‐I algorithm (Katoh and Standley 2013). Alignments were trimmed with trimAl v1.4.rev15 (Capella‐Gutiérrez, Silla‐Martínez, and Gabaldón 2009) in gappyout mode, and sequences that contained > 60% gaps after trimming were removed. The aligned sequences were concatenated per genome, filling in gaps when a gene was absent from the alignment. The final concatenated alignment contained 208 representative MAGs from the Black Sea and 1354 representative genomes from GTDB, and included 8944 aligned amino acids.

We constructed a maximum‐likelihood phylogenetic tree with IQ‐TREE v2.1.2 (Nguyen et al. 2015) using 1000 ultrafast bootstraps (Minh, Nguyen, and Haeseler 2013) and model selection (Kalyaanamoorthy et al. 2017) best‐fit model (LG + F + R10) chosen based on the Bayesian Information Criterion (BIC). Visualisation was done in Interactive Tree of Life (iTOL) (Letunic and Bork 2024).

### 4.6 Search for Homologues of Targeted Genes and Their Abundance Profile

Homologues of the targeted genes were identified by blastp+ (v. 2.7.1) (Camacho and Madden 2023) in all proteins predicted on the scaffolds by Prodigal with e‐value < 1 × 10^−30^ and percentage of identity > = 30%. Query sequences of targeted protein‐coding genes were the following: Tes protein of *Methanococcus aeolicus* Nanakai‐3 (accession number ABR56159.1), GrsA protein of *Sulfolobus acidocaldarius* (accession number WP_011278400.1), elbD protein of *Myxococcus xanthus* (accession number ABF88003.1), agps protein of *Myxococcus xanthus* (accession number ABF89845.1), Gms protein of *Thermococcus guaymasensis* DSM 11113 (accession number AJC70771.1).

Bubble plots for the visualisation of hits in MAGs across the water column were done with Python 3 and Matplotlib in JupyterLab (https://jupyter.org/).

The script jgi_summarize_bam_contig_depths that comes with MetaBAT2 (Kang et al. 2019) was used to extract average depth (i.e., number of mapped reads per base pair) for each scaffold that contained hits from the BAM files, and depth was normalised per 1 × 10^8^ mapped reads in each sample. The CAT annotation of the scaffold, or the BAT annotation of the MAG if the scaffold was binned, was used for taxonomic classification of the hits.

The normalised average depth profiles of a scaffold that are based on all versus all mappings can be used to compare it to other scaffolds, but they cannot be summed to aggregate profiles from a certain taxon, as reads from one sample may map to similar strains in different samples, and thus be counted multiple times when summed. This leads to an overestimation of the abundance of taxa that are present in multiple samples. When aggregating the abundance of taxa, we thus summed normalised average depth only for those mappings from which the scaffold was assembled. For example, the summed normalised average depth of Halobacteriota with *grsA* homologues at 500 m depth only includes the scaffolds with hits assembled from that sample, and similarly for 1000 m depth and 2000 m depth. As no scaffolds of Halobacteriota with *grsA* hits were found in other samples, their summed normalised average depth is 0, even if the abundance profile of individual scaffolds (which is based on the all versus all mappings) may show presence in some of these samples.

## Author Contributions

**Dina Castillo Boukhchtaber:** investigation, writing – original draft, writing – review and editing, data curation, methodology, validation, visualization, conceptualization. **F. A. Bastiaan von Meijenfeldt:** conceptualization, methodology, visualization, writing – review and editing, data curation, validation. **Diana X. Sahonero Canavesi:** writing – review and editing, methodology, data curation. **Denise Dorhout:** writing – review and editing, methodology, data curation, formal analysis. **Nicole J. Bale:** validation, supervision, data curation, writing – review and editing, methodology. **Ellen C. Hopmans:** data curation, supervision, validation, methodology. **Laura Villanueva:** conceptualization, investigation, funding acquisition, writing – original draft, writing – review and editing, validation, project administration, supervision, data curation.

## Conflicts of Interest

The authors declare no conflicts of interest.

## Supporting information


**Data S1.** Supplementary Figure.

**Data S2.** Supplementary Tables.

**Data S3.** Supporting Information.

## Data Availability

The 16S rRNA gene amplicon reads (raw data) can be accessed in the NCBI Sequence Read Archive (SRA) under BioProject IDs PRJNA423140, and PRJNA649254‐57. The Black Sea MAGs are available in Zenodo at https://doi.org/10.5281/zenodo.10346513. The phylogenetic tree file and iTOL annotation files can be found in Zenodo at https://doi.org/10.5281/zenodo.12200551. all the Supporting Information have been deposited at ZENODO (DOI: https://doi.org/10.5281/zenodo.14651037) Link: https://urldefense.com/v3/__https://zenodo.org/records/14651037__;!!N11eV2iwtfs!qEOpTyg1IZMCJmv9t8OdX_IicWRC5RmlBYVyRfwwJAawv2PqsAc‐ORyNgdGVlUm5inWETfBfolr7EsNdT2z0n1BEmKKspWBDrVc\$.
